# Cardiovascular Effects of Herbal Products and Their Interaction with Antihypertensive Drugs—Comprehensive Review

**DOI:** 10.3390/ijms25126388

**Published:** 2024-06-09

**Authors:** Kinga-Ilona Nyulas, Zsuzsánna Simon-Szabó, Sándor Pál, Márta-Andrea Fodor, Lóránd Dénes, Margit Judit Cseh, Enikő Barabás-Hajdu, Bernadett Csipor, Juliánna Szakács, Zoltán Preg, Márta Germán-Salló, Enikő Nemes-Nagy

**Affiliations:** 1 Doctoral School of Medicine and Pharmacy, George Emil Palade University of Medicine, Pharmacy, Science, and Technology of Târgu Mureş, 540139 Târgu Mureș, Romania; 2 Department of Pathophysiology, Faculty of Medicine, George Emil Palade University of Medicine, Pharmacy, Science, and Technology of Târgu Mureş, 540139 Târgu Mureș, Romania; 3 Department of Laboratory Medicine, Department of Transfusion Medicine, Medical School, University of Pécs, 7622 Pécs, Hungary; 4 Department of Laboratory Medicine, Faculty of Medicine, George Emil Palade University of Medicine, Pharmacy, Science, and Technology of Târgu Mureş, 540139 Târgu Mureș, Romania; 5 Department of Anatomy and Embryology, Faculty of Medicine, George Emil Palade University of Medicine, Pharmacy, Science, and Technology of Târgu Mureş, 540139 Târgu Mureș, Romania; 6 Master Program of Nutrition and Dietetics, George Emil Palade University of Medicine, Pharmacy, Science, and Technology of Târgu Mureş, 540139 Târgu Mureș, Romania; 7 Department of Cell Biology and Microbiology, Faculty of Pharmacy, George Emil Palade University of Medicine, Pharmacy, Science, and Technology of Târgu Mureş, 540139 Târgu Mureș, Romania; 8 Department of Biophysics, Faculty of Medicine, George Emil Palade University of Medicine, Pharmacy, Science, and Technology of Târgu Mureş, 540139 Târgu Mureș, Romania; 9 Department of Family Medicine, Faculty of Medicine, George Emil Palade University of Medicine, Pharmacy, Science, and Technology of Târgu Mureş, 540139 Târgu Mureș, Romania; 10 Department of Internal Medicine III, Faculty of Medicine, George Emil Palade University of Medicine, Pharmacy, Science, and Technology of Târgu Mureş, 540139 Târgu Mureș, Romania; 11 Department of Chemistry and Medical Biochemistry, Faculty of Medicine in English, George Emil Palade University of Medicine, Pharmacy, Science, and Technology of Târgu Mureş, 540139 Târgu Mureș, Romania; eniko.nemes-nagy@umfst.ro

**Keywords:** phytotherapy, hypertension, antihypertensive drugs, herb–drug interaction, herbal products

## Abstract

Hypertension is a highly prevalent population-level disease that represents an important risk factor for several cardiovascular complications and occupies a leading position in mortality statistics. Antihypertensive therapy includes a wide variety of drugs. Additionally, the potential antihypertensive and cardioprotective effects of several phytotherapy products have been evaluated, as these could also be a valuable therapeutic option for the prevention, improvement or treatment of hypertension and its complications. The present review includes an evaluation of the cardioprotective and antihypertensive effects of garlic, Aloe vera, green tea, Ginkgo biloba, berberine, ginseng, Nigella sativa, Apium graveolens, thyme, cinnamon and ginger, and their possible interactions with antihypertensive drugs. A literature search was undertaken via the PubMed, Google Scholar, Embase and Cochrane databases. Research articles, systematic reviews and meta-analyses published between 2010 and 2023, in the English, Hungarian, and Romanian languages were selected.

## 1. Introduction

Hypertension affects more than one billion people worldwide, and it is a major risk factor for various cardiovascular diseases. It is a leading cause of death globally, claiming 10.8 million lives in 2019 [1]. In adults, the latest data suggest a hypertension prevalence of 30% in the developed countries [2]. It is estimated that 1.8 billion adults between the ages of 30 and 79 are likely to be diagnosed with high blood pressure, and two-thirds of them live in low- and middle-income countries [3]. In addition to positive lifestyle changes, most people with high blood pressure require regular, daily pharmaceutical therapy. The overall aim is to prevent or delay the development of complications for as long as possible [4]. Besides the available medicines, various herbal teas and natural products emerged as part of integrative medicine, which, in addition to rational phytotherapy, includes a healthy diet and lifestyle, as well as other effective complementary therapies for hypertensive patients [5].

The preference for phytotherapy products is justified by the benefits of long-term safe dosing, reduced risk of side effects and adverse drug interactions, and the absence of dysbacteriosis [6]. A healthy lifestyle (including a diet rich in fruits and vegetables, restricted salt and alcohol intake, weight reduction, regular physical activity, and smoking cessation) also has beneficial effects on blood pressure [7]. If lifestyle changes are not successful at normalizing blood pressure, monotherapy is usually recommended first in Stage 1 hypertension. If it fails, combination therapy should be implemented. Vasodilators, such as angiotensin-converting enzyme (ACE) inhibitors, angiotensin receptor blockers (ARBs), calcium channel blockers (CCBs) or beta-blockers, thiazide-like diuretics (leading to elimination of water and minerals) are currently used in the treatment of hypertension. Multidrug therapy is often required to achieve target blood pressure values, but certain combinations should be avoided (such as ACE inhibitors with ARBs, or beta-blockers with nondihydropyridine CCBs). Single-pill combinations of different drugs in low doses with synergistic effects decrease the risk of adverse effects and improve patient compliance. This is a safer and more efficient strategy for treating Stage 2 or 3 hypertension compared to high-dose monotherapy, which often leads to adverse effects. The most frequently used combinations are ACE inhibitors/ARBs with diuretics (thiazide or thiazide-like), or ACE inhibitors/ARBs with CCBs [4,8].

Herbal products (such as green tea, garlic, Aloe vera, Ginkgo biloba, berberine, ginseng, Nigella sativa, Apium graveolens, thyme, cinnamon, ginger, hawthorn, and pomegranate) are included in the composition of several highly accessible dietary supplements with cardioprotective effects, and these supplements can also be used in combination with certain antihypertensive drugs to improve therapeutic efficacy.

The most frequently used phytotherapy products in the treatment of hypertension exhibit their cardioprotective effect by different mechanisms of their bioactive substances. These effects include antioxidant, anti-inflammatory, and antithrombotic properties, vasodilation, enzyme induction or inhibition, or synergistic effects with the medication [6].

Adverse effects of herb–drug interactions might be caused by drug plasma levels increasing beyond the minimum toxic concentrations; thus, patients taking medication with narrow therapeutic windows are more exposed to harmful effects. Young infants, elderly people, and pregnant women represent populations that are vulnerable to potential toxicity. In addition, impaired liver and/or kidney function, certain genetic diseases, or patients with transplanted organs might require monitoring during combination therapy. Polypragmasia, which is frequently associated with phytotherapy, leads to an increased risk of drug–herb interactions and adverse effects. Potential contamination of herbal products with impurities of heavy metals or pesticides, depending on the location where they grow; misidentification of the plants; and the presence of secondary metabolites represent inherent hazards. The geographical region of origin, the time of harvesting, and the extraction methods used can lead to considerable differences in commercially available herbal products. In spite of these limitations, a relatively reduced number of herbal products are contraindicated to be used in combination with conventional medicines taken in regular doses [9].

The aim of the present study is to review the cardioprotective mechanisms of the included phytotherapy products and their interplay with antihypertensive drugs.

## 2. Materials and Methods

Data collection was performed between July 2023 and February 2024, and articles related to antihypertensive phytotherapy products published between 2010 and 2023 in the English, Hungarian, and Romanian languages were reviewed. A literature search of PubMed, Google Scholar, Embase, and Cochrane databases was performed using the English, Romanian, and Hungarian keywords included in Table 1. The publications were filtered based on the following criteria: open access articles published in the stated time interval and written in the above-mentioned languages. Primarily, research articles, secondarily systematic reviews, meta-analyses and, finally, review articles were considered. 

To analyze the mechanism of action of the included products/substances, the selection of clinical research articles and of animal experimental articles was prioritized. Data relating to the most accessible and commonly used phytotherapy products were processed, and these were determined to be green tea, garlic, Ginkgo biloba, Aloe vera, ginseng, berberine, Nigella sativa, Apium graveolens, thyme, cinnamon, and ginger. The exclusion criteria were related to publication in other languages, paid articles, letters to the editor, case reports, conference summaries, abstracts, and doctoral dissertations. The included records have been processed by prioritizing papers with high subject numbers and reproducible methodologies.

Every author of this paper is an advanced speaker of all three languages mentioned in Table 1. The authors followed the strategy of distributing the subtopics on each herb to different researchers (except the drug–herb interactions, which were written by the same author for all medicinal plants) to avoid matching papers from different databases. Additionally, other two authors checked all the citations and corresponding references to avoid duplicates.

The search for articles on drug–herb interactions was performed in PubMed and Embase databases with the following outcome: 5139 articles were selected in the first step according to the aforementioned criteria, followed by a rigorous selection based on the title and the abstract, which reduced this number to 105 full-text articles; from these, 40 were included as references. Additionally, searching for herb–drug interactions in the DrugChecker application involved a combination of the following keywords: “name of the medicinal herb” and “name of antihypertensive drug” and “interaction”. Duplicates were eliminated via a DOI search; some publications without a DOI were double-checked manually.

The figures were created using R statistical software (R Core Team, 2024, Vienna, Austria), version 4.4.0, with the “ggplot2” package, and Microsoft Excel was used to create the tables.

## 3. Results

The search of four different databases resulted in 49 articles from PubMed, 48 articles from Google Scholar, 23 articles from Embase, and 6 articles from Cochrane Library. Each herbal product will be discussed with regard to its bioactive form/forms and cardiovascular effects, followed by its interaction with antihypertensive drugs.

### 3.1. Green Tea

#### 3.1.1. Bioactive Substances, Cardiovascular Effects

Green tea is extracted from the Camellia sinensis plant and it is consumed worldwide. In Asian countries, it is considered a traditional remedy for various conditions. The beneficial effects of this plant are attributed to its polyphenolic compound content with flavonoid-like properties, particularly catechin compounds. Among the most important catechins are epigallocatechin gallate, epicatechin gallate, epigallocatechin, and epicatechin, which are listed as the active ingredients with a positive effect on health [9]. In vitro studies have demonstrated that catechins derived from green tea have cardioprotective properties. The underlying mechanism is attributed to the antioxidant, antithrombotic actions of catechins, and their capacity to improve endothelial dysfunction and to reduce vascular inflammation [10,11].

In vivo experimental animal studies confirmed the improvement of endothelial dysfunction and hypertension through the effects of catechins on nitric oxide (NO) production and vasodilation. Several observational longitudinal studies have examined the relationship between blood pressure (BP) reduction and green tea consumption, with controversial results. According to the reports of various authors, the consumption of green tea may decrease BP [12,13], while other studies could not provide evidence to confirm these findings [14]. Based on the meta-analysis of 24 studies involving a total of 1697 subjects, Xu et al. concluded that regular consumption of green tea results in a statistically significant reduction in both systolic and diastolic blood pressure (DBP) [15]. Al-Shafei et al. analyzed the cardiovascular effects of regular, medium-term green tea consumption in 200 study subjects. The study concluded that green tea consumption decreased heart rate, as well as systolic and DBP [16]. Arazi et al. investigated the short-term (three weeks administration) cardiovascular effects of regular (twice daily) consumption of green tea and could not demonstrate an improvement in heart rate, nor BP [17]. The meta-analysis of Liu et al. suggested a significant, mild antihypertensive effect of green tea, which developed after regular long-term (over at least twelve weeks) consumption [13]. Dietary supplements containing green tea extracts have blood pressure-lowering, anti-inflammatory, and antioxidant effects. Additionally, they reduce insulin resistance in obese and hypertensive individuals [18,19].

#### 3.1.2. Mechanisms of Herb–Drug Interactions

Co-administration with green tea may significantly decrease the plasma concentrations and urinary excretion of lisinopril. The mechanism of interaction has not yet been identified; however, it may involve impairment of intestinal absorption of lisinopril by green tea catechins. In a study involving ten healthy volunteers (young Japanese adults), administration of a single 10 mg oral dose of lisinopril with 200 mL aqueous solution of green tea extract containing approximately 300 mg of epigallocatechin gallate (EGCG) resulted in 71% and 66% decreases in the mean lisinopril peak plasma concentration (Cmax) and systemic exposure, respectively, compared to administration with water. The time taken to reach the peak lisinopril concentration (Tmax) and renal clearance of lisinopril were not significantly altered [20].

Similarly, co-administration with green tea may significantly decrease the plasma concentrations of nadolol. The mechanism of interaction is not yet fully elucidated and may involve inhibition of organic anion-transporting polypeptides (OATP)1A2-mediated uptake of nadolol in the bowel by green tea catechins. It has already been shown by in vitro studies that green tea catechins inhibit the activity of OATP1A2 and OATP2B1 expressed in the apical side of the intestinal epithelium in humans [21]. The effects of green tea extract and epigallocatechin-3-gallate (EGCG) on the pharmacokinetics of nadolol, markedly reducing its bioavailability, were also studied in rats [22].

In a randomized, three-phase crossover study involving thirteen healthy volunteers (Japanese adults), administration of a single 30 mg oral dose of nadolol following repeated consumption of green tea (700 mL/day for 14 days) resulted in decreases of 85% in nadolol peak plasma concentration (Cmax) and systemic exposure (AUC) compared to administration with water. The renal clearance of nadolol was not altered. Green tea also markedly reduced the effects of nadolol on systolic blood pressure (SBP) [23,24,25]. A few years later, the same team of researchers demonstrated that only a single cup of green tea administered concomitantly or one hour before nadolol intake can substantially decrease the plasma concentration of nadolol by inhibiting its intestinal absorption [26]. As the available data on these moderate interactions of green tea with lisinopril or nadolol may be influenced by the cultural background of the subjects, it is advisable to inform and counsel patients to limit their consumption of green tea and green tea extracts during treatment with lisinopril or nadolol.

Animal experiments proved that green tea—more precisely, its catechins—affects the pharmacokinetics of amlodipine, as well. Due to this herb–drug interaction, the Cmax and the AUC0–t of amlodipine increased, while the Tmax of amlodipine decreased in rats pretreated with epigallocatechin gallate, and the metabolic half-life was also prolonged by EGCG, showing that both EGCG and green tea extracts could inhibit the metabolism of amlodipine by inhibiting the activity of cytochrome P450 (CYP3A) enzyme [27].

### 3.2. Garlic

#### 3.2.1. Bioactive Substances, Cardiovascular Effects

Numerous clinical studies confirm that garlic (Allium sativum) is associated with reduced blood pressure, even in a dose-related manner; this effect is mainly due to its organosulfur compounds like allicin and diallyl disulfide. Allicin elevates reduced glutathione (GSH) concentration in the erythrocytes and increases the genetic expression of several antioxidant enzymes (superoxide dismutase (SOD), catalase (CAT), and glutathione peroxidase (GPX)), thus reducing oxidative stress and protecting endothelial cells and cardiomyocytes from apoptosis. Allicin induces vasodilation by enhancing NO release and increasing the levels of cyclic AMP and cyclic GMP. Allicin inhibits LDL-cholesterol uptake by the macrophages in the vessel wall, preventing atherosclerosis. Allicin’s antihypertensive efficacy was described to be similar to that of enalapril in clinical studies [28]. In a placebo-controlled study, the SBP and DBP of patients taking 2 × 400 mg tablets of garlic extract improved significantly after 15 weeks of therapy [29].

The examination of garlic-derived protein hydrolysate revealed its inhibitory effect on angiotensin-converting enzyme (ACE) activity, reducing the concentrations of angiotensin II. Thus, garlic is synergistic with ACE inhibitor drugs or with angiotensin receptor blockers. While the side-effect profile of clinically used ACE inhibitors varies (including symptoms such as leukopenia, skin rash, cough, and taste disorder), small doses of the natural compounds from garlic extracts do not exert any side effects; moreover, they may be potent antioxidants [30].

#### 3.2.2. Mechanisms of Herb–Drug Interactions

Studies on garlic compounds and their use for medical purposes in humans have not been associated with alterations in the activity of key drug-metabolizing enzymes, such as CYP1A2, CYP2D6, or CYP3A4. It can be safely emphasized that these compounds are unlikely to modulate the pharmacokinetics and pharmacodynamics of the co-administered drugs. However, the potential for garlic to be involved in transporter-based drug interactions has not been adequately addressed in human studies, and preclinical evidence is limited. Despite this gap, current human in vivo studies do not suggest clinically remarkable metabolism-based interactions between garlic and antihypertensive drugs cleared by the CYP1A2, CYP2D6, CYP2E1, or CYP3A4 enzymes [31,32].

At the same time, other animal and human studies have proven the existence of herb–drug interactions, which are induced by various mechanisms. Assessing the impact of diallyl trisulfide (DATS) in rats, a major garlic-derived component, on the pharmacokinetics of nifedipine administered orally and intravenously, led to the conclusions that diallyl trisulfide notably increased the oral bioavailability of nifedipine, elevating the oral exposure of nifedipine in rats, likely by influencing the intestinal metabolism of nifedipine. Consequently, caution is advised when considering the concurrent use of diallyl trisulfide-containing supplements with nifedipine to prevent potentially toxic plasma concentrations of nifedipine [33].

The interaction between garlic supplements and antihypertensive medications like losartan, valsartan, furosemide, and hydrochlorothiazide involves complex dynamics in intestinal absorption and plasma concentrations. The presence of garlic supplements may impact the bioavailability of these drugs, particularly valsartan and losartan, due to their extensive liver metabolism and enterohepatic cycling. This interaction could lead to decreased intestinal absorption, a lower fraction of dose absorbed (Fabs), and altered plasma concentrations. The impact of garlic supplements on intestinal transporters is influenced by the specific active compound, the permeability model, and the level of transporter expression [34].

Some herb–drug interactions can have beneficial effects on the body, such as the synergism between carvedilol and garlic. An animal study showed the pathophysiological impact of interaction between aged garlic extract (AGE) or S-allyl-L-cysteine (SAC, an active component of AGE) and carvedilol on rats with isoproterenol-induced myocardial dysfunction. High doses of AGE and SAC, administered for 3 weeks alone or with carvedilol, significantly reduced serum lactate dehydrogenase (LDH) and creatine kinase MB (CK-MB) activities and increased endogenous antioxidants in heart tissue, indicating that high doses of AGE and SAC, combined with carvedilol, can synergistically prevent structural and functional damage of the myocardium [35]. A similar experiment demonstrated that there was an increase in the cardioprotective effect of carvedilol when it was used concomitantly with garlic oil or diallyl disulfide (the primary component of garlic oil) [36].

The association of atenolol and AGE or SAC enhances cardioprotection by preserving membrane integrity and reducing oxidative stress, to which atenolol contributes as a β-blocker by reducing the cardiac workload, while AGE and SAC contribute through their antioxidant effects, as well as through workload reduction. The weaker cardiovascular protection of atenolol benefits from AGE and SAC’s antioxidant actions. The combination effectively decreases SOD levels and thiobarbituric acid-reactive substances (TBARS), indicating reduced oxidative damage and enhanced prevention of isoproterenol-induced myocardial injury. Even though both AGE and SAC individually offer cardioprotection, their synergy with atenolol holds promise for more effective prevention in susceptible patients [37]. Co-administration of garlic with propranolol can also be effective for the benefit of the patients, yielding positive outcomes. Animal experiments have confirmed that the bioavailability of propranolol doubled and its half-life tripled when rats were given garlic. The practical positive consequences of this include a decrease in SBP, cholesterol, triglycerides (which are particularly beneficial in hypertensive animals struggling with myocardial stress) and glucose levels, as well as observed reductions in fluid intake and body weight. However, due to the decrease in propranolol clearance and elimination rate constants, the possibility of propranolol toxicity should not be overlooked, especially during repeated administration, as drug accumulation is possible [38,39].

### 3.3. Aloe Vera

#### 3.3.1. Bioactive Substances, Cardiovascular Effects

Aloe vera is a plant that has been used for thousands of years and contains a variety of bioactive substances, such as anthraquinones (barbaloin, chromones), which have a diuretic effect in high doses; anti-inflammatory compounds (salicylic acid, sterols, hormones like auxin and gibberellins); immunomodulator carbohydrates; and several enzymes and vitamins (vitamin A, E, C). From a biological perspective, its antioxidant components are of paramount importance. The variations in the compound content of the plant are related to the geographical region of origin of the plant [40,41].

Metabolic syndrome is often associated with diabetes, dyslipidemia, obesity, and hypertension. The cumulative risk factors contribute to a poorer prognosis in these patients. Aloe vera is an effective phytotherapy product for this condition, improving carbohydrate balance and reducing insulin resistance [42]. Yaqoob et al. investigated the effect of Aloe vera on blood pressure versus mono-dose diclofenac, administered daily, using a hypertensive experimental rat model. The study concluded that Aloe vera did not have a significant influence on blood pressure, while diclofenac led to increased values in the examined animals [43]. The available human studies on the blood pressure-lowering effect of Aloe vera are limited. Most studies focus on emodin, which inhibits substrate phosphorylation and phenylephrine-induced phosphorylation of myosin light-chains. Through this mechanism, Aloe vera inhibits vasoconstriction caused by phenylephrine, endothelin-1, and 5-hydroxytryptamine [44]. Several in vitro studies have shown that emodin was effective in inhibiting the proliferation of human vascular smooth muscle cells, but not that of human vascular endothelial cells. Vascular smooth muscle plays an important role in the development of atherosclerosis and hypertension and, thus, atherosclerotic plaque formation and restenosis can be prevented via emodin-eluting stents [45].

#### 3.3.2. Mechanisms of Herb–Drug Interactions

Current evidence suggests that Aloe vera does not interact adversely with commonly prescribed antihypertensive medications. However, it is important to consider the inherent laxative properties of Aloe vera and, consequently, its potential to decrease the intestinal transit time or reduce the absorption of co-administered drugs. A reduction in the effectiveness or efficacy of drugs could have negative consequences, particularly for the ones with a narrow therapeutic index [46].

### 3.4. Ginkgo Biloba

#### 3.4.1. Bioactive Substances, Cardiovascular Effects

Ginkgo biloba is a member of the Ginkgoaceae family. It is recognized by its white fruits and distinctive leaves, which are considered to be beneficial for medicinal purposes [47]. Ginkgo biloba improves circulation and venous status and has numerous other beneficial effects: it offers hepatoprotection, acts as an antitumor agent and antioxidant, has an antianxiety effect, enhances mental activity and cognitive performance, and alleviates the symptoms of dementia. So far, consumption of Ginkgo biloba has not been associated with altered kidney function. The plant improves the consequences of ischemia–reperfusion injuries by affecting lipid metabolism. Due to the solubility of the active substances of Ginkgo biloba, modern therapy typically uses its standardized extract of acetone–water mixture [48]. In a study on Ginkgo biloba extract, researchers could successfully block platelet-derived growth factor (PDGF) synthesis through inhibition of tyrosine kinase. Ginkgo biloba extract consumption could reduce platelet aggregation without disrupting blood clotting. The leaves of this plant contain ginkgolides (diterpene lactones), bilobalides (sesquiterpenes), and flavonoids (quercetin and kaempferol). These substances improve blood circulation through their vasodilator effect, especially in the brain circulation, skin, ocular microcirculation, and muscles. In the elderly, Ginkgo biloba extract is used to prevent dementia. EGb 761 (the conventional name of the most-studied Ginkgo biloba leaf extract) is a highly concentrated and standardized extract regarding the total content of flavonoids (kaempferol) and terpenoids (bilobalides and ginkgolides). Unfortunately, many Ginkgo biloba products on the market contain non-standardized extracts that do not have the phytochemical profile of EGb 761 [49,50].

The cardioprotective effects of Ginkgo biloba are supported by an increasing number of studies reporting vasodilation and antihypertensive effects as the underlying mechanism. After three months of regular daily intake, Ginkgo biloba reduced SBP by 6% and DBP by 21% [51].

#### 3.4.2. Mechanisms of Herb–Drug Interactions

The scientific literature describes various interactions between Ginkgo biloba and certain antihypertensive medications, leading to a decrease in their effectiveness in animal trials. Dong B. et al. investigated the effects of Ginkgo biloba on the pharmacokinetics of losartan and its metabolite EXP3174 (losartan carboxylic acid) in rat liver microsomes. Their results indicated that Ginkgo biloba could increase the systemic exposure of losartan while decreasing the systemic exposure of EXP3174 by inhibiting its metabolism. It is important to mention that the clinical efficacy of EXP3174, a potent, selective non-peptide angiotensin II (AT1) receptor antagonist, is about 10-fold of that of its parent drug, losartan [52]. The potential influence of Ginkgo biloba and ginseng on main drug-metabolic cytochrome P450 enzyme (CYP450) was also investigated in another animal study, which consisted of repeated administration of Sailuotong, a fixed combination of Ginkgo biloba, ginseng, and saffron for vascular dementia. The study demonstrated different activities on multiple isoforms of CYP450 enzyme in rats: an inductor effect on CYP1A2 and CYP2C11, but with significant inhibition of CYP3A1 and CYP3A2. These observed impacts were primarily attributed to the individual or cooperative actions of ginseng and Ginkgo biloba constituents [53]. There is better-documented evidence that Ginkgo biloba increases talinolol blood concentration [54].

### 3.5. Berberine

#### 3.5.1. Bioactive Substances, Cardiovascular Effects

Berberine is an alkaloid found in various medicinal herbs such as barberry, goldenseal, Oregon grape, common mahonia, and turmeric. It has a rich history of utilization spanning over 3000 years in Chinese and Ayurvedic traditional medicine. Initially used to treat gastrointestinal infections, berberine demonstrates various positive impacts on health. Notably, it has the potential to lower BP, serum cholesterol, and blood glucose. In specific abnormal cardiac conditions, like arrhythmias, berberine may serve as a beneficial adjunctive therapy, with the efficacy of amiodarone, but no side effect [55]. At cellular level, berberine modulates glucose utilization in liver and fat cells, promoting glycolysis, while inhibiting oxidation processes in mitochondria. The compound is also an inhibitor of alpha-glucosidase, resulting in diminished disaccharidase enzyme activity and reduced glucose transport within intestinal epithelial cells. Moreover, berberine plays an important role in reducing insulin resistance, contributing to a decrease in the waist circumference and waist-to-hip ratio by facilitating fatty tissue redistribution. Despite its therapeutic potential, berberine consumption is associated with gastrointestinal side effects.

The hypoglycemic effect of berberine is similar to that of metformin, as it reduces levels of glycated hemoglobin, fasting glucose, and postprandial glucose. Yin et al. described how berberine diminishes levels of reactive oxygen species and programmed cell death triggered by elevated glucose concentrations in endothelial cells through the activation of adenosine monophosphate-activated protein kinase activity and nitric oxide synthesis [56].

Berberine is recognized for its efficacy in reducing triglyceride and LDL-cholesterol levels; nonetheless, certain patients have reported a reduction in HDL-cholesterol levels. Additionally, gastrointestinal side effects, including diarrhea, constipation, bloating, and abdominal pain, have been documented. Fortunately, these side effects can be alleviated through dose adjustments [57].

A meta-analysis has shown that lifestyle intervention combined with berberine in the treatment of hypertension reduces blood pressure better than lifestyle changes or placebo alone, and berberine combined with an oral antihypertensive also reduces blood pressure better than the same antihypertensive alone [55]. The related mechanism can be attributed to berberines’ effect on inhibiting oxidative stress and inflammation in hypertension and diabetes, improving renal hemodynamics.

According to Soltani et al., berberine consumption did not exert a significant blood pressure-lowering effect [58].

#### 3.5.2. Mechanisms of Herb–Drug Interactions

Earlier studies have established a similarity between the pharmacokinetic profiles of losartan and EXP3174 in rats and humans, justifying the use of rats to explore potential herb–drug interactions. Research investigating the impact of berberine on the metabolic stability of losartan using rats revealed significant changes in the pharmacokinetic profiles of losartan and EXP3174 due to berberine. The plasma concentration of losartan was increased, while that of EXP3174 was decreased, aligning with inhibited losartan metabolism and reduced EXP3174 concentration. All these can be attributed to the inhibition of CYP3A4 or CYP2C9, as losartan is a P-glycoprotein (P-gp) substrate, and it is metabolized by CYP3A4 and CYP2C9 [59]. A two-phase randomized-crossover clinical study in humans also demonstrated the inhibitory effects of berberine on CYP2D6, CYP3A4, and CYP2C9, showing the herb–drug interaction between losartan and berberine [60].

In the context of herb–drug interactions, it is also interesting to follow the effect of drugs on herbs, like the change in pharmacokinetics of berberine due to irbesartan. After oral administration in rats, berberine has low bioavailability, fast absorption, and slow metabolism rates due to secondary absorption and entero-hepatic circulation. When co-administered with irbesartan, as both are lipid-soluble and interact with CYP3A4 and P-gp, irbesartan enhances berberine’s bioavailability and absorption by inhibiting P-gp in the intestinal tract, extending its contact with CYP3A4, while berberine increases irbesartan’s concentration but has minimal influence on irbesartan metabolism. This co-administration elevates blood concentrations of both berberine and irbesartan compared to separate oral administrations [61].

### 3.6. Ginseng

#### 3.6.1. Bioactive Substances and Cardiovascular Effects

Ginseng, taxonomically classified in the angiosperm division, dicotyledon class, and Apiales order within the family Araliaceae, belongs to the Panax genus, which is renowned for several subgenera. The Greek term “panax”, translating to “cure-all”, elucidates its extensive historical use in medicine for millennia in both Asia and North America owing to its diverse bioactive compounds [62].

In general, the term encompasses American ginseng (Panax quinquefolius) and Asian/Korean ginseng (Panax ginseng). Among other compounds (like phytosterols, polysaccharides, fatty acids, peptides, tanning agents, vitamins B_1_, B_2_, and minerals), the effectiveness of Panax is attributed to ginsenoside, a dammarane-type triterpene saponin. Beyond its complexity as a biomolecule, it has been observed to be able to be metabolized into various bioactive compounds, which have anti-inflammatory and antioxidant effects, and also influence apoptosis and angiogenesis [63,64,65].

It is imperative to underscore that ginseng sourced from diverse geographical regions manifests distinct biological effects. Furthermore, variations in the concentration and type of ginsenoside, coupled with the processing method, impact its biological effects and the concentration of ginsenosides [66,67,68].

One extensively researched effect of ginseng pertains to its influence on the cardiovascular system, with a wealth of references and well-documented studies available in both Asian and North American sources, including materials in languages other than English [62].

Research findings in English-language literature trace back to the 1960s and 1970s, when Wood and colleagues were pioneers in demonstrating, through animal experimental models, the blood pressure-reducing effect of Panax ginseng extract [67,69].

As a result, ginsenoside Rg3, due to its specific influence in this regard, emerges prominently as one of the extensively investigated and well-documented components within Panax [69,70,71].

The blood pressure-lowering effect attributed to ginsenoside Rg3 is linked to the vasodilation it induces, coupled with an increased release of NO. Additionally, the activation of calcium-dependent potassium (K) channels has been observed [67,72].

The increased release of NO in endothelial cells, attributed to general saponin, is an important contributing factor. This is facilitated by an augmented intracellular influx of Ca^2+^. The ensuing activation of calcium-dependent K-channels on the cell membrane leads to the influx of Ca^2+^ into endothelial cells. The elevated Ca^2+^ concentration stimulates NO synthesis in the endothelium, resulting in the relaxation of smooth muscle cells and subsequent vasodilation. The efficacy of blood pressure reduction is dependent on both the concentration and presence of saponins [69].

An additional potential explanation for the decrease in blood pressure is the inhibitory effect exerted by ginseng on the release of catecholamines. Kim’s noteworthy observation underscores the blood pressure-normalizing effect of ginseng, concurrently highlighting its antidiabetic and anticarcinogenic properties [72].

Mucalo et al. documented a blood pressure-elevating effect, particularly in normalizing hypotension. This phenomenon could be attributed to alterations in rheological parameters or the adaptive characteristics of baroreflexes [73].

Research on phytotherapeutic agents encounters challenges, as it is insufficient to individually scrutinize the components and their mechanisms of action. The overall effect requires the simultaneous presence of its complex components [74]. Unfortunately, there is a phenomenon known as “ginseng abuse syndrome”, which is primarily characterized by insomnia, hypertension, edema, and diarrhea [39,65].

#### 3.6.2. Mechanisms of Herb–Drug Interactions

A comprehensive pharmacokinetic analysis, carried out in rats with a fixed-dose triple combination of fimasartan/amlodipine/hydrochlorothiazide, revealed potential herb–drug interactions with red ginseng extract. Notably, no clinically relevant interactions have been observed between the three active substances, except for a mild influence of fimasartan on the urinary excretion of hydrochlorothiazide. When administered alone or in combination with red ginseng extract, fimasartan exhibited consistent profiles with no alterations in Cmax, AUClast, AUCinf, T1/2, and Mean Residence Time (MRT) values. Similarly, in the case of hydrochlorothiazide, it was apparent that no herb–drug interaction could be detected. Only amlodipine exhibited a delayed Tmax following multiple red ginseng extract administrations, and this was attributed to altered absorption kinetics due to decreased intestinal permeability [75]. Several animal studies indicated that red ginseng has minimal impact on the pharmacokinetics of amlodipine, as well as on that of losartan and its active metabolite EXP-3174, in rats [76,77].

No in vivo herb–drug interaction was observed between red ginseng extract (administered orally or intravenously) and valsartan, possibly due to the low plasma concentration of protopanaxadiol (PPD)-type ginsenosides. Additionally, even at high plasma concentrations, ginsenoside Rc did not significantly affect the pharmacokinetics of valsartan, which was primarily attributed to its high protein binding and limited distribution in the liver [78]. In a study involving fifteen healthy young male volunteers, the potential for drug interactions of red ginseng extract was investigated using a cocktail of probe substrates (losartan, dextromethorphan, pitavastatin, omeprazole, midazolam, and caffeine), but the result showed no clinically significant differences. Red ginseng did not demonstrate relevant interactions with the same drug-metabolizing enzymes (CYP1A2, CYP2C9, CYP2C19, CYP2D6, and CYP3A4) and transporters (ex. OATP1B1) in in vitro studies, either. However, it is important to note that these findings may not apply to other forms of red ginseng, as their content may differ from the tested product [79].

It is worth noting that resistance to furosemide induced by ginseng has been reported. The exact mechanism is unknown, but positivity has been shown for dechallenge and rechallenge methods used in pharmacovigilance, and the timing of self-medication with high concentrations of ginseng-based herbal supplements and the onset of symptoms (edema and hypertension) unresponsive to increased doses of furosemide strongly suggest a possible herb–drug interaction, and patients treated with furosemide or other loop diuretics should avoid the use of ginseng supplements. Pharmacokinetic studies in animal models have shown that long-term administration of a medicinal product containing ginseng may lead to increased retention of furosemide in the body, affecting its absorption, distribution, metabolism, and excretion; however, there were no observed changes in the efficacy of furosemide. Related pharmacokinetic interaction studies have not yet been reported in humans. Mechanisms regulating furosemide excretion include renal and hepatic glucuronidation metabolism and excretion via multidrug resistance protein 4 (MRP4) and organic anion transporters (OATs). These mechanisms may potentially be affected by ginsenosides [80].

### 3.7. Celery (Apium graveolens)

#### 3.7.1. Bioactive Substances and Cardiovascular Effects

Apium graveolens (celery) is a spice herb widely used in Ayurvedic traditional medicine. It exhibits a wide variety of pharmacological effects, including antihypertensive, antioxidant, anti-obesity, hepato-, gastro- and cardioprotective, antidiabetic, cholesterol-lowering, anti-inflammatory, antiaggregant, diuretic, and anticancer properties. The extracts obtained from this plant contain several phytochemical compounds and secondary metabolites such as tocopherols, carotenes, flavonoids, terpenoids, steroids, alkaloids, glycosides, amino acids, tannins, saponins, anthraquinones, phenylpropanoids, coumarins, polysaccharides, and organic acids [81,82].

Mostly flavonoids (apigenin, luteolin, kaempferol), phthalides (butylphthalide) and monoterpenes might be responsible for its antihypertensive effect, enhancing blood vessel relaxation [83]. A significant decrease in blood pressure values could be observed in a study carried out on elderly hypertensive patients after administration of celery extract for three months [84].

In two recent triple-blind, placebo-controlled clinical trials, celery seed extract capsules administered four times per day for one month proved to be efficient in significantly lowering systolic and diastolic blood pressure of young, middle-aged, and elderly hypertensive patients as an adjuvant of the current antihypertensive medication, without any adverse effect [85,86].

Experimental studies on isolated rat aorta demonstrated that celery seed extract induces vasorelaxation by inhibition of ryanodine receptor and/or inositol triphosphate (IP_3_) pathway and reduces intracellular Ca^2+^, reduces myosin light-chain kinase activity through diacylglycerol activating protein kinase C (DAG-PKC)-dependent pathway, and decreases intracellular Ca^2+^ through blocking receptor-operated Ca^2+^ channels (ROCCs), inhibition of calcium influx into vascular smooth-muscle cells and voltage-dependent potassium channels [83].

#### 3.7.2. Mechanisms of Herb–Drug Interactions

Despite a thorough and careful review of the scientific literature, no specific data (studies) could be found regarding potential herb–drug interactions between antihypertensive medicinal products and celery, even though the blood pressure-lowering effect of celery is well-known and proven.

Celery seeds have an inhibitory effect on phase I enzymes, specifically CYP 450s. They induce the activity of phase II enzymes, such as glutathione S-transferase. Additionally, celery seed inhibits efflux transporters, particularly P-glycoprotein (P-gp).

Apigenin, a predominant flavonoid found in celery, can function both as a substrate and as an inhibitor of the CYP3A4 enzyme, which is responsible for the metabolism of more than 30% of the medicinal products. In vitro studies have confirmed that apigenin irreversibly inhibits CYP3A4 enzyme activity. On the other hand, celery seeds act as inductors on phase II enzymes, but they may interact with other drugs at their site of action (through targeting, synergistic effects, or various other mechanisms) [87,88].

### 3.8. Nigella Sativa

#### 3.8.1. Bioactive Substances and Cardiovascular Effects

Nigella sativa (Black cumin/Black seed) belongs to the family Ranunculacee. Its seeds have been used as both spices and medicinal plants since ancient times.

Its oil, which is pressed from its seeds, contains linoleic acid and glycerides of oleic acid. Its active ingredients are thymoquinone, thymohydroquinone, thymol, carvacrol, nigellidine, nigellicidine, α-hederin. In addition to its numerous active ingredients, it also has a high nutritional value; it contains important amounts of proteins, fatty acids, amino acids, vitamins, and minerals.

The oil of Nigella sativa has many clinically proven medicinal properties: it reduces oxidative stress and inflammation and has antioxidant, immunomodulatory, antitumoral, neuroprotective, antimicrobial, antihypertensive, cardioprotective, antidiabetic, and gastroprotective effects. In traditional medicine, its use extends to the treatment of asthma, bronchitis, rheumatic diseases, headache, back pain, anorexia, eczema, and arterial hypertension.

According to the results of clinical studies, it reduces systolic and diastolic blood pressure through several mechanisms. By antagonizing the effects of angiotensin II, it raises LVDP (left ventricular end-diastolic pressure), reduces MAP (mean arterial pressure) and HR (heart rate), provides protection against oxidative stress by raising SOD and CAT activities, lowers MDA (malondialdehyde) levels, and has diuretic effects, calcium channel-blocking, and cardiac depressant properties [89,90].

Black cumin reduces sympathetic activity, improves the lipid profile (reducing serum total cholesterol, LDL-cholesterol, and triglycerides, and raising HDL-cholesterol), and increases nitrite oxide production [91]. It decreases IL_6_, TNF-α, and IL_2_ levels, and acts as a natural antioxidant [92]. 

#### 3.8.2. Mechanisms of Herb–Drug Interactions

A study investigating how Nigella sativa (black cumin) affects the pharmacodynamics and pharmacokinetics of amlodipine used hypertensive rats treated with either amlodipine, Nigella sativa, or Nigella sativa + amlodipine. The combined treatment resulted in better control of blood pressure (compared to administration of the herb or drug alone), and Nigella sativa did not significantly alter the pharmacokinetic parameters of amlodipine (Cmax, AUC0-t, Kel, terminal elimination half-life). At the same time, Nigella sativa reduced heart rate as an additional benefit [93]. Similar synergism was observed with losartan because Nigella sativa enhanced the antihypertensive effect of losartan rats. The plasma concentration of losartan increased with Nigella sativa, indicating a potential herb–drug interaction, likely due to the herb’s inhibitory impact on CYP3A4 and CYP2C9 enzyme activity. Therefore, dosage adjustments are necessary [94]. The effect of black cumin on the pharmacokinetics and pharmacodynamics of metoprolol was also investigated. Although both metoprolol and Nigella sativa alone reduced blood pressure in L-NAME (N(gamma)-nitro L-arginine methyl ester)-induced hypertensive rats, the drug–herb combination had a stronger blood pressure-lowering effect on systolic, diastolic and mean arterial pressures. Regarding heart rate, it was lowest when only Nigella sativa was administered, with the combination with metoprolol providing a less satisfactory improvement in heart-rate reduction [90,95].

### 3.9. Cinnamomum Verum

#### 3.9.1. Bioactive Substances and Cardiovascular Effects

Cinnamomum spice is produced from the bark of certain tree species from the genus Cinnamomum (*Cinnamonum cassia*, *C. burmanni*, *C. loureiroi* (*Saigon cinnamon*), *C. verum*, *C. citriodorum*). The active ingredients of Cinnamomum are eugenol, cinnamaldehyde, camphor, cinnamic acetate, copane, coumarin, and numerous essential oils.

The active ingredients of cinnamon increase the uptake of glucose into cells and improve insulin sensitivity. Cinnamomum polyphenols lower blood sugar levels and reduce systolic and diastolic blood pressure. One of the antihypertensive mechanisms of cinnamon involves its potential to inhibit ACE, as demonstrated in vitro. In an experimental study, the percentage of ACE inhibition was compared in sheep lung, testis, and kidney tissues using a methanolic extract of Cinnamon zeylanicum (10:1) as a natural inhibitor, with a captopril standard drug serving as the control. The ACE inhibitory property of cinnamon has been proven, with the inhibition being the greatest in kidney tissue [96,97]. The methanol extract of Cinnamomum zeylanicum demonstrated both acute and chronic antihypertensive effects in L-NAME-induced hypertensive rats. Acutely, it significantly reduced mean arterial blood pressure, primarily through vasodilation. Chronically, it alleviated hypertension, reduced cardiac hypertrophy, decreased vasoconstriction, and enhanced arterial wall compliance by increasing nitric oxide production and preventing endothelial damage [98].

Various components of cinnamon, such as epicatechin, catechin, and procyanidin B2, can inhibit the generation of advanced glycation products (AGEs) [96]. Eugenol and conifer-aldehyde improve blood circulation and inhibit blood clotting, and their effectiveness is comparable to that of acetylsalicylic acid. Antitumor, anti-inflammatory, analgesic, antibacterial, antiviral, cytoprotective, neuroprotective, immunomodulatory, and anti-tyrosinase effects have been described [99]. 

Cinnamon has antiatherogenic properties, as it has a protective effect on endothelial cells. In mouse thoracic aortic isolates, cinnamaldehyde reduced contraction, improved endothelial NO production, and reduced AGEs-dependent endothelial damage. Daily consumption of 1.3 or 6 g of cinnamon reduced serum LDL-cholesterol, triglyceride, and total cholesterol levels [100].

In an in vitro study, eugenol prevented the formation of reactive oxygen species (ROS). It has been experimentally proven that cinnamon extract inhibits LDL oxidation in RAW264.7 macrophage cells in mice. The benefits of cinnamon on lipid metabolism are achieved through certain enzymes and genes, as it enhances the activity of enzymes known for their potential to inhibit lipid peroxidation of SOD and CAT, respectively, through the expression of haemeoxygenase-1 (HO-1). Cinnamon also reduces fasting blood sugar, body mass index, and glycosylated hemoglobin (HbA1c) [101] and reduces CRP levels [102]. Inhibition of platelet activity and antithrombotic effect (thromboxane A_2_ inhibition) are key to the cardioprotective effects of cinnamon [103]. By inhibiting angiogenesis, it has an anti-inflammatory, anticancerogenic effect. Its effect on hypertension is questionable, and some studies have not been able to prove its hypotensive effect [104]. It seems to have an antiarrhythmic effect by reducing ventricular ectopic beat and ventricular tachycardia [105].

Side effects of cinnamon use include gastrointestinal adverse effects (e.g., stomachache, nausea) and allergic reactions. Due to its coumarin content, it may cause liver damage in sensitive individuals. It is unsafe to use cinnamon during pregnancy and breastfeeding, and it may cause hypoglycemia in diabetic patients [106].

#### 3.9.2. Mechanisms of Herb–Drug Interactions

Cinnamon is frequently encountered in human consumption through food and herbal treatments. However, the potential interactions of its components, cinnamaldehyde and methoxy cinnamaldehyde, with drugs metabolized by CYP2A6 remain poorly understood. These interactions with CYP2A6 are intricate, involving various and complex mechanisms such as time-dependent inhibition of CYP2A6 substrate (like nicotine) metabolism (at least two mechanisms contribute to this), multiple-ligand binding, apoprotein modification, and heme (partial) inactivation and degradation. Predictions regarding herb–drug interactions suggest that prolonged exposure to cinnamon may result in significant interactions, particularly with nicotine [107].

The scientific literature revealed no significant interactions between cinnamon and antihypertensive medications, even though cinnamon has both systolic and diastolic blood pressure-lowering effects.

### 3.10. Wild Thyme (Thymus serpyllum)

#### 3.10.1. Bioactive Substances and Cardiovascular Effects

Wild thyme is an aromatic herb widely used as a spice and in traditional medicine, especially in the Mediterranean region. Its essential oils contain phenolic monoterpenes (thymol, carvacrol), while its infusion contains several polyphenols like phenolic acids (rosmarinic acid, caffeic acid) and flavonoids (apigenin, luteolin, quercetin and eriocitrin). Thyme extracts exhibit vasorelaxant properties due to the presence of rosmarinic acid and induce upregulation of heme oxygenase 1, leading to carbon monoxide release, due to caffeic and rosmarinic acids, which explains the antihypertensive effect of this medicinal plant. Furthermore, thyme has anti-inflammatory, antioxidant, antiaggregant, antidiabetic, and antimicrobial properties [108,109,110].

Thyme is generally a safe herb, but it has some potential adverse effects: it is not recommended as adjuvant in pregnant women because of the potential risk of miscarriage and it might cause irritation of the mucous membranes in sensitive individuals due to the phenolic monoterpenes present in its composition. A study on a small number of hypertensive patients showed a significant decrease in SBP and DBP in the group treated with thyme extract for two weeks as an adjuvant to current antihypertensive medication compared to subjects only receiving drug therapy [111].

Similar findings were described in animal experiments. A study on spontaneously hypertensive rats demonstrated a significant decrease in SBP, DBP and peripheral resistance after bolus administration of 100 mg/kg body weight thyme extract [108].

Another research group obtained similar findings in rats, and the effects were additionally confirmed by biochemical changes, histological and ultrastructural imaging [112].

#### 3.10.2. Mechanisms of Herb–Drug Interactions

According to a clinical study carried out on hypertensive patients in Iraq, the concomitant use of antihypertensive medication and thyme resulted in a significant decrease in DBP compared to the values obtained with drug treatment alone [111]. In a clinical study conducted across five hospitals in Ethiopia, the efficacy and safety of the simultaneous use of antihypertensive drugs with herbal products, including thyme, were evaluated. Out of a total of 365 patients included in the study, only 15 used thyme while the others used different herbal products. Although no details were given regarding the duration of the antihypertensive treatment or the dosage, it was stated that no interactions were experienced between conventional drugs and thyme [113].

### 3.11. Ginger

#### 3.11.1. Bioactive Substances and Cardiovascular Effects

Ginger (Zingiber officinale) is a spice and is one of the most widely used medicinal plants in the world, mostly in Asian countries. Ginger contains high amounts of phenolic compounds (paradol, zingerone, gingerols, and shogaols), which have powerful antioxidant effects and reduce lipid peroxidation and, thus, atherosclerosis, which is involved in the development of high blood pressure. Certain phenolic compounds from its composition also exhibit vasodilator properties by increasing NO plasma concentration. Cholinergic effects of phenolic compounds are also notable, reducing BP by activation of metabotropic and muscarinic acetylcholine receptors of blood vessel epithelial cells. Additionally, calcium channel blocking and ACE inhibiting effects of ginger have been described in animal experiments. Ginger also has antidiabetic, antilipidemic, anticancer and anti-inflammatory properties [114,115,116,117].

Ginger showed relevant antihypertensive effects, especially in patients under fifty years of age, when administered in relatively high doses (exceeding 3 g per day) for up to two months [114].

A study on 120 hypertensive patients, mostly females, showed significantly lower blood pressure values after one month of drinking ginger juice once a day with or without antihypertensive therapy compared to a control group taking only hypertensive drugs [118].

In a large cross-sectional study on 4628 participants, the researchers concluded that a 2–4 g of daily ginger intake could prevent population-level chronic diseases, including hypertension and other cardiovascular diseases [115].

No studies showed any toxicity as a result of ginger administration; it is generally very well tolerated, although high doses might cause mild gastrointestinal manifestations (nausea, dyspepsia, gastric irritation) as potential adverse effects. Regarding pharmacological interactions, ginger should be used with precaution in combination with aspirin and cumarinic anticoagulants as it may increase the risk of bleeding [114,119].

#### 3.11.2. Mechanisms of Herb–Drug Interactions

According to an animal study investigating the pharmacokinetic and pharmacodynamic effect of ginger on amlodipine in hypertensive rats, although both amlodipine and ginger administered alone lowered BP, the antihypertensive effect was greater when they were administered together. Ginger also showed clear action on the pharmacokinetics of amlodipine. This herb–drug interaction provides additional pharmacodynamic benefits during concomitant use [120].

Another study confirmed that ginger enhances the blood pressure-lowering effect of losartan in L-NAME-induced hypertensive rats. In the presence of ginger, the hypotensive effect of losartan was more pronounced, with significantly increased plasma concentrations of the drug. Ginger altered the pharmacokinetic parameters of losartan, such as Cmax, AUC0-t, and t1/2, likely by inhibiting the CYP enzymes responsible for losartan elimination. This led to an increased elimination half-life and higher plasma concentrations of losartan. Additionally, ginger alone also reduced BP, suggesting a potential pharmacodynamic interaction. Further studies are needed to explore these interactions with different dose levels [121]. In an in vitro environment, ginger has had a competitive inhibitory effect on CYP2C19 enzyme, (without significant influence on the other CYP isoenzymes), so the possibility of pharmacokinetic interaction with drugs metabolized by CYP2C19 must be considered [122].

The mechanisms of the cardiovascular effects exerted by the selected herb products are summarized in Table 2 and Table 3. The herb–drug interaction is summarized in Table 4.

## 4. Discussion

Although there are many smart tools for identifying herb–drug interactions (e.g., Epocrates or Natural Medicines), some of them are unavailable to those located in the EU, while others are not free, or only provide information on interactions between conventional drugs. DrugChecker (https://www.drugs.com/drug_interactions.html, accessed on 3 January 2024) is an application designed to assess Drug Interaction Classifications, providing a systematic framework for evaluating potential interactions. Developed by Drugs.com, (https://www.drugs.com/support/editorial_policy.html, accessed on 3 January 2024) DrugChecker aligns with the platform’s mission to be the preeminent online resource for accurate, up-to-date drug and health-related information, by delivering independent, objective and comprehensive data in a format that is accessible to both patients and healthcare professionals. The classification in four categories (major, moderate, minor and unknown) built into the application serves as a useful, practical and pragmatic guideline for evaluating the significance of drug–drug, herb–drug and food-drug interactions, but their clinical relevance to a specific individual can be challenging to determine.

The most common mechanism of action in the cardiovascular system of the studied herbal products is enhancing nitric oxide synthesis, which causes vasodilation, followed by the inhibition of angiotensin-converting enzyme II and the antioxidant effect of certain medicinal plants.

**Figure 1:**
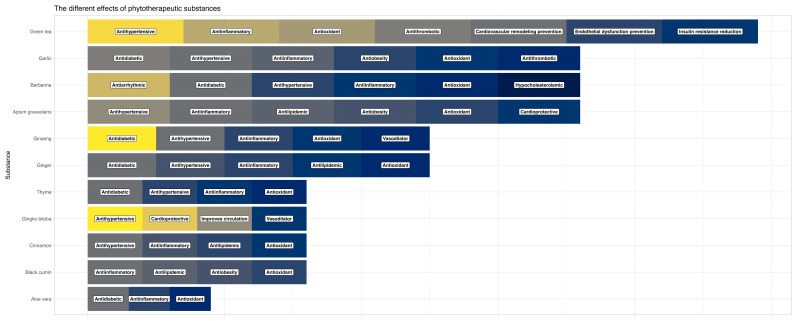
Cardiovascular effects of the medicinal plants.

The cardiovascular effects of the different medicinal plants are presented in Figure 1.

**Figure 2:**
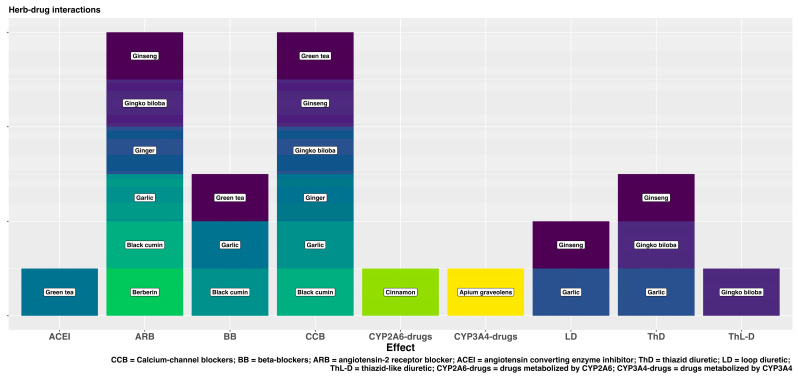
Drug–herb interactions.

The most frequent drug–herb interaction is the influence of herbal products on the cytochrome p450 system in the liver, which is responsible for the metabolization of the antihypertensive drugs, increasing their plasma concentration and the risk of potential toxicity. Figure 2 shows the most important interactions of the studied herbal products with the major groups of antihypertensive drugs.

Effective management of interactions between medications and herbs involves several key strategies. Health professionals need to receive comprehensive education and training on herbal medicines, which is often lacking in conventional medical curricula. Raising awareness among both healthcare providers and the public is crucial to encourage open communication about the use of herbal products. Patients should be encouraged to provide complete information about their medication history, diet, and any herbal products they are using or intend to use. Additionally, the existing standardized reporting systems for suspected herb–drug interactions should be used by both healthcare professionals and consumers within pharmacovigilance frameworks. Widespread consumer education is necessary to ensure individuals recognize the importance of communicating openly with their healthcare providers about their use of both traditional and pharmaceutical treatments. It would also be ideal to improve research strategies regarding herb–drug interactions to provide highly relevant and valid evidence. Overall, an integrated approach involving education, awareness, reporting mechanisms and research is necessary for the effective management of medication-herb interactions [123].

For the safe application of medicinal herbs, it is very important that they are administered on medical recommendation, or at least the patient should inform their treating physicians about which herbal preparations they are consuming. According to the scientific literature, it is very common for people to use multiple medicinal herbs at the same time, sometimes with opposite effects, and often they consume them before surgical procedures without considering it important to inform their doctors, thereby exposing themselves to great risk. They often do not consider that medicinal herbs can also have harmful, unwanted side effects. At the same time, most healthcare professionals do not routinely ask their patients whether they are using other products for therapeutic purposes alongside their medications. If it were possible to alter the everyday practice for doctors and pharmacists to ensure that they specifically mention phytotherapeutic agents when inquiring about a patient’s treatment, it would significantly improve communication and there would be a greater chance of revealing/identifying other preparations being used. The necessity of rational phytotherapy needs to be recognized [5,124,125,126].

The originality of this review lies in the limited availability of comprehensive studies related to the use of phytotherapy agents as supplementary or potential antihypertensive treatments and the assessment of herb–drug interaction. The process of selecting multilingual bibliography adds to the quality of the present article.

This review is subject to limitations that should be considered. Firstly, a subset of the referenced sources reports controversial information, largely stemming from disparities in the composition of specific phytotherapy products. Secondly, the included studies reported the effect of the selected phytotherapy products based on observations conducted over varying durations. Thirdly, the variability and, occasionally, the small number of subjects included in the studies represent additional limitations. In the latter case, a notable difference in the reported observations could be explained by differences in the ethnicity of the populations in which some studies were performed. Additionally, in the case of certain phytotherapy agents, a reduced number of subjects were available, and for certain included substances, the amount of available and reliable published data is limited.

## 5. Conclusions

The reviewed literature indicates that there is no consensus on the use and evaluation of phytotherapy agents and their efficacy in patients with hypertension. The majority of publications emphasize the beneficial effects of various dietary supplements, but many studies report conflicting results.

Almost all the phytotherapeutic agents investigated by us, including green tea, garlic, ginseng, Ginkgo biloba, berberine, Apium graveolens (celery), cinnamon, and Nigella sativa (black cumin), generally exhibit blood pressure-lowering effects. Additionally, Ginkgo biloba, green tea, cinnamon, celery, and black cumin have demonstrated cardioprotective properties, contributing positively to cardiac health.

Our results have observed herb–drug interactions with several antihypertensive drugs: green tea, Ginkgo biloba, ginseng, and garlic can interact with several diuretics, beta-blockers, calcium channel blockers, ACE inhibitors, sartans, and angiotensin II receptor antagonists. Berberine interacts specifically only with angiotensin II receptor type 1 antagonists. Cinnamon affects drugs metabolized by CYP2A6, whereas Apium graveolens affects drugs metabolized by CYP3A4. Aloe vera and thyme do not interact with antihypertensive drugs. Concomitant use of the studied herbs with conventional antihypertensive medications generally resulted in beneficial effects for patients, enhancing both the cardioprotective potential and the antihypertensive efficacy. The notable exceptions to this were green tea and Ginkgo biloba, which did not consistently provide these benefits. It is crucial for healthcare providers to be aware of these potential interactions and consider them when prescribing conventional medications alongside these herbs to avoid adverse effects and maximize therapeutic benefits.

Medical decisions on drug therapy will only be accurate and adequate if based on systematically identified, critically evaluated, and applied scientific information on the safety, efficacy, and interaction properties of herbal medicines.

Based on growing evidence, traditional medicine can be integrated in the modern concept of patient-oriented therapy, as an adjuvant of conventional drug-based treatment. Phytotherapy should be introduced according to medical advice, not based on self-therapy, to achieve the most potent combinations and to avoid potentially harmful interactions with the current medication. 

## Figures and Tables

**Table 1:** Keywords used for literature search in the three languages.

English	Hungarian	Romanian
antihypertensive	antihipertenzív	antihipertensiv
dietary supplement	táplálékkiegészítő	supliment alimentar
effect	hatás	efect
herb	gyógynövény	plantă medicinală
hypertension	magasvérnyomás	hipertensiune arterială
interaction	interakció	interacțiune
mechanism of action	hatásmechanizmus	mecanism de acțiune
phytotherapy	fitoterápia	fitoterapie

**Table 2:** Cardiovascular effects (I) of the main phytotherapy products.

	Actions	NO ↑	Antioxidant Enzymes ↑	LDL chol. Uptake ↓	ACE-II ↓	Platelet Aggregation ↓/Antithrombotic	Inhibition of Oxidation	CKDC
Products								
***Green tea***		x						
***Garlic***		x	x		x			
***Aloe vera***								
***Ginkgo biloba***						x		
***Berberine***		x		x			x	
***Ginseng***		x						x
***Celery (Apium graveolens)***								
***Nigella sativa***		x	x	x	x		x	x
***Cinnamomum verum***		x	x	x	x	x		
***Wild thyme (Thymus serpyllum)***		x	x			x		
***Ginger***		x			x			x

Abbreviations: NO: nitric oxide, LDL chol.: low-density lipoprotein cholesterol, ACE-II: angiotensin-converting enzyme II, CDKC: calcium-dependent potassium (K) channel. X-marks the presented action, symbols ↑ and ↓ represent the stimulating or inhibiting effect of the substance on the presented action.

**Table 3:** Cardiovascular effects (II) of the main phytotherapy products.

	Actions	HR ↓	Inhibitiory Effect	GSH ↑	cAMP ↑	cGMP ↑	Other Effects
Products							
***Green tea***		x	CYP 3A				
***Garlic***				x	x	x	
***Aloe vera***			substrate phosphorylation & phenylephrine-induced phosphorylation				Vasoconstriction ↓
			vascular smc proliferation				diuretic
***Ginkgo biloba***			CYP 3A				
			tyrosine kinase				
			PDGF syntesis				
***Berberine***			alpha-glucosidase				diuretic
							glucose transport
***Ginseng***							chatecolamines ↓
							normalizing hypotension
***Celery (Apium graveolens)***			ryanidone receptor				diuretic
			IP3 pathway				VGKC
							myosine light chaine kinase activity ↓
***Nigella sativa***		x					diuretic
***Cinnamomum verum***			tyrosine kinase				AGEs ↓
			angiogenesis				
***Wild thyme (Thymus serpyllum)***							upregulation of heme oxygenase-1
***Ginger***							

Abbreviations: HR: heart rate; CYP 3A: cytochrome P 450; family 3; subfamily A; GSH: reduced glutathione; cAMP: cyclic adenosine monophosphate; cGMP: cyclic guanosine monophosphate; PDGF: platelet-derived growth factor; IP3: inositol triphosphate; AGEs: advanced glycation end products; smc: smooth muscle cell; VGKC: voltage-gated potassium channels. X-marks the presented action, symbols ↑ and ↓ represent the stimulating or inhibiting effect of the substance on the presented action.

**Table 4:** Effects and mechanisms of drug–herb interaction.

Herb	Drug	Effect	Mechanism
***Green tea***	lisinopril	plasma concentration ↓	
	nadolol	plasma concentration ↓	OATP1A2 and OATP2B1 inhibition, ↓ absorbtion, (OATP)1A2-mediated uptake ↓
	amlodipine		cytochrome P450 system inhibition
***Garlic***	nifedipine	intestinal metabolism ↑, plasma concentration ↑	
	valsartan and losartan	intestinal absorption ↓, plasma concentration ↓	intestinal transporters↓
	carvedilol	LDH ↓, CK-MB ↓, antioxidant ↑	
	atenolol	cardiac workload↓	oxidative stress↓
	propranolol	SBP ↓, cholesterol, tryglicerides, glucose ↓	
***Aloe vera***	general effect	intestinal transit time ↓, intestinal absorption ↓	
***Ginkgo biloba***	losartan/ EXP3174	plasma concentration ↑/plasma concentration ↓	
	talinolol	plasma concentration ↑	
***Berberine***	losartan/ EXP3174	plasma concentration ↑/plasma concentration ↓	inhibition of CYP2D6, CYP3A4 and CYP2C9
	irbesartan	plasma concentration ↑	
***Ginseng***	fimasartan/amlodipine/hydrochlorothiazide	no interaction	
	furosemide	resistance	
***Celery (Apium graveolens)***	non	no interaction	
***Nigella sativa***	amlodipine	HR ↓	
	losartan	plasma concentration ↑	inhibition of CYP3A4 and CYP2C9 enzyme activity
	metoprolol	enhancing effect	
***Cinnamon***	nicotine		time-dependent inhibition of CYP2A6
***Wild thyme***	non	no interaction	
***Ginger***	amlodipine	enhancing effect	
	losartan	plasma concentration ↑	inhibition of CYP enzymes
	clopidogrel		competitive inhibitory effect on CYP2C19 enzyme

Abbreviations: (OATP)1A2/2B2: human organic anion-transporting polypeptide 1A2/2B2; LDH: lactate dehydrogenase; CK-MB: creatine kinase muscle–brain; SBP: systolic blood pressure; HR: heart rate; CYP2D6: cytochrome P 450, family 2, subfamily D, member 6; CYP3A4: cytochrome P 450, family 3, subfamily A, member 4; CYP2C9: cytochrome P 450, family 2, subfamily C, member 9; CYP2A6: cytochrome P 450, family 2, subfamily A, member 6; CYP2C19: cytochrome P 450, family 2, subfamily C, member 19; CYP: cytochrome P 450; EXP3174: losartan carboxylic acid. Symbols ↑ and ↓ represent the stimulating or inhibiting effect of the substance on the presented drugs metabolization and bioavailability.
